# Phylogenomics Reshuffles the Eukaryotic Supergroups

**DOI:** 10.1371/journal.pone.0000790

**Published:** 2007-08-29

**Authors:** Fabien Burki, Kamran Shalchian-Tabrizi, Marianne Minge, Åsmund Skjæveland, Sergey I. Nikolaev, Kjetill S. Jakobsen, Jan Pawlowski

**Affiliations:** 1 Department of Zoology and Animal Biology, University of Geneva, Geneva, Switzerland; 2 Department of Genetic Medicine and Development, University of Geneva, Geneva, Switzerland; 3 Department of Biology, University of Oslo, Oslo, Norway; University College Dublin, Ireland

## Abstract

**Background:**

Resolving the phylogenetic relationships between eukaryotes is an ongoing challenge of evolutionary biology. In recent years, the accumulation of molecular data led to a new evolutionary understanding, in which all eukaryotic diversity has been classified into five or six supergroups. Yet, the composition of these large assemblages and their relationships remain controversial.

**Methodology/Principle Findings:**

Here, we report the sequencing of expressed sequence tags (ESTs) for two species belonging to the supergroup Rhizaria and present the analysis of a unique dataset combining 29908 amino acid positions and an extensive taxa sampling made of 49 mainly unicellular species representative of all supergroups. Our results show a very robust relationship between Rhizaria and two main clades of the supergroup chromalveolates: stramenopiles and alveolates. We confirm the existence of consistent affinities between assemblages that were thought to belong to different supergroups of eukaryotes, thus not sharing a close evolutionary history.

**Conclusions:**

This well supported phylogeny has important consequences for our understanding of the evolutionary history of eukaryotes. In particular, it questions a single red algal origin of the chlorophyll-c containing plastids among the chromalveolates. We propose the abbreviated name ‘SAR’ (Stramenopiles+Alveolates+Rhizaria) to accommodate this new super assemblage of eukaryotes, which comprises the largest diversity of unicellular eukaryotes.

## Introduction

A well resolved phylogenetic tree describing the relationships among all organisms is one of the most important challenges of modern evolutionary biology. A current hypothesis for the tree of eukaryotes proposes that all diversity can be classified into five or six putative very large assemblages, the so-called ‘supergroups’ (reviewed in [1] and [2]). These comprise the ‘Opisthokonta’ and ‘Amoeboza’ (often united in the ‘Unikonts’), ‘Archaeplastida’ or ‘Plantae’, ‘Excavata’, Chromalveolata’, and ‘Rhizaria’. The supergroup concept as a whole, however, has been shown to be only moderately supported [3] and the evolutionary links among these groups are yet to be confirmed. These uncertainties may be due to the limited amounts of available data for the most parts of the eukaryotic diversity. In particular, only a small fraction of the unicellular eukaryote diversity [4] has been subject to molecular studies, leading to important imbalances in phylogenies and preventing researchers to reliably infer deep evolutionary relationships.

The supergroup Rhizaria [5] is particularly interesting for testing different possible scenarios of eukaryote evolution. This assemblage has only recently been described and is based exclusively on molecular data; nevertheless it is very well supported in most phylogenies [3]. It includes very diverse organisms such as filose testate amoeba, cercomonads, chlorarachniophytes (together, core Cercozoa), foraminifers, plasmodiophorids, haplosporidians, gromiids, and radiolarians (see [2] for an overview or [6]–[11]). In opposition to Rhizaria, the monophyly of Chromalveolata is far from being undisputed (see [12], or [3], [13]–[15]). Chromalveolates were originally defined by their plastid of red algal origin that (when present) is believed to have arisen from a single secondary endosymbiosis [16]. This supergroup encompasses many ecologically important photosynthetic protists, including coccolithophorids (belonging to the haptophytes), cryptophytes, diatoms, brown seaweeds (together, the chromists) and dinoflagellates (which form together with ciliates and apicomplexans the alveolates) [17], [18].

Using a phylogenomic approach we recently confirmed the monophyly of Rhizaria and addressed the question of its evolutionary history [19]. The analyses of 85 concatenated nuclear protein sequences led to two potential affiliations with other eukaryotes. According to the first hypothesis, Rhizaria was sister group to an excavate clade defined by *G. lamblia*, *T. vaginalis*, and Euglenozoa. The second hypothesis suggested that Rhizaria are closely related to stramenopiles, which form together with alveolates, haptophytes, and cryptophytes the supergroup of chromalveolates. Besides our study, the branching pattern between Rhizaria and other supergroups has been specifically evaluated only by Hackett et al. (2007), who reported a robust relationship between Rhizaria and members of the chromalveolates.

Here, we further address the phylogenetic position of Rhizaria within the eukaryotic tree using an extensive multigene approach. For this purpose, we have carried out two expressed sequence tag (EST) surveys of rhizarian species: an undetermined foraminiferan species belonging to the genus *Quinqueloculina* (574 unique sequences, Accession Numbers: EV435154-EV435825) and *Gymnophrys cometa* (628 unique sequences, Accession Numbers: EV434532-EV435153) (Cienkowski, 1876), a freshwater protist that has been shown to be part of core Cercozoa [20]. Using novel EST datasets for two rhizarians [21], [22] and data from publicly available protists (TBestDB; http://tbestdb.bcm.umontreal.ca/searches/login.php), we constructed a taxonomically broad dataset of 123 protein alignments amounting to nearly 30000 unambiguously aligned amino acid positions. Our superalignment includes several representatives for all described eukaryotic supergroups. Our results show an unambiguous relationship between Rhizaria and stramenopiles, confirming the hypothesis we had previously proposed and suggesting the emergence of a new super assemblage of eukaryotes that we propose to name ‘SAR’ (stramenopiles+alveolates+Rhizaria).

## Results

### Single-gene analyses and concatenation

49 eukaryotic species representatives of all five current supergroups for which large amounts of data are available were selected. We identified 123 genes (see Table S1) that fulfilled the following criteria: 1) at least one of the four rhizarian species as well as at least one member of unikonts, plants, excavates, alveolates, and stramenopiles were present in every single-gene alignment; 2) the orthology in every gene was unambiguous on the base of single-genes bootstrapped maximum likelihood (ML) trees. This second criterion is particularly important in multigene analyses in order to avoid the mixture of distant paralogs in concatenated alignments, because it would dilute the true phylogenetic signal by opposing strong mis-signals, thus preventing the recovering of deep relationships [23]. Similarly, it is essential to detect and discard putative candidates for endosymbiotic gene transfer (EGT) or Horizontal Gene Transfer (HGT). Hence, we submitted each of our single-gene alignments to ML reconstructions with bootstrap replications and systematically removed sequences that displayed ambiguous phylogenetic positions for both paralogy and gene transfers. For example, we found few cases where *B. natans* and *G. theta* sequences actually corresponded to genes encoded in the nucleomorph genome of these species. This restrictive procedure allowed us to have a set of 123 single-gene alignments, each of them containing at least one rhizarian species, with only orthologous sequences, and virtually no gene transferred either from a plastid or from a foreign source.

One possible approach to analyse such a dataset is to build a supermatrix that is formed by the concatenation of individual genes (for a review see [23]). After concatenation, our final alignment contained 29908 unambiguously aligned amino acid positions. Overall, we observed an average missing data of 39% but these sites were not uniformly distributed across taxa (see Tables S2 and S3 for more details). However, several studies have demonstrated that the phylogenetic power of a dataset remains as long as a large number of positions are still present in the analysis [24]–[27]. For example, Wiens [26], [27] demonstrated that the inclusion of highly incomplete taxa (with up to 90% missing data) in model-based phylogenies, such as likelihood or Bayesian analysis, could cause dramatic increases in accuracy.

### Phylogenetic position of Rhizaria

**Figure 1:**
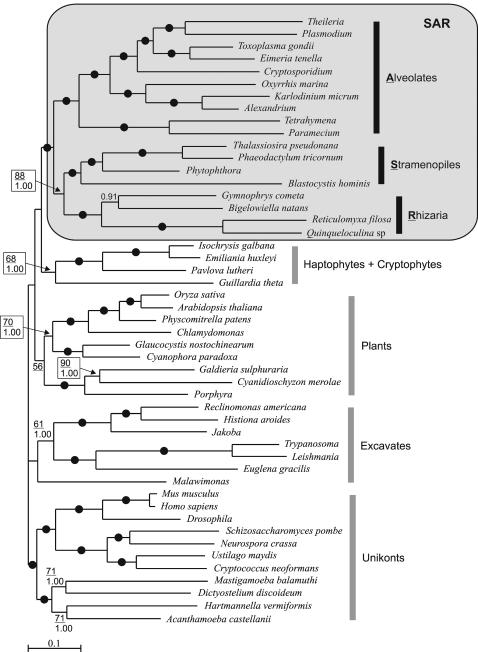
Best maximum likelihood tree of eukaryotes found using TREEFINDER, with 10 starting trees obtained with the global tree searching procedure. Numbers at nodes represent the result of the bootstrap analysis (underlined numbers; hundred bootstrap pseudoreplicates were performed) and Bayesian posterior probabilities. Black dots represent values of 100% bootstrap support (BP) and Bayesian posterior probabilities (BiPP) of 1.0. Nodes without numbers correspond to supports weaker than 50% BP and 0.8 BiPP.

The ML and Bayesian trees inferred from the complete alignment (Figure 1; see also Figure S1 and S2) recover a number of groups observed previously and are in most aspects congruent with global eukaryotic phylogenies published recently [14], [28], [29]. A monophyletic group uniting Metazoa, Fungi, and Amoebozoa (altogether the unikonts) was robustly supported (100% bootstrap support, BP; 1.0 Bayesian posterior probability, BiPP); green plants, glaucophytes, and rhodophytes came together, albeit only weakly supported (56% BP; this node was not recovered in the Bayesian analysis, see Figure S2); a group composed of haptophytes and cryptophytes, as well as excavates (without Malawimonas that failed to consistently branch with the other excavates species) received only moderate supports for their union in the ML inference (68% and 61% BP, respectively) but 1.0 BiPP. Finally, alveolates, stramenopiles, and Rhizaria all formed monophyletic groups with 100% BP and 1.0 BiPP. Although most of the recognized eukaryotic supergroups are recovered in our analyses, the relationships among them are generally not well resolved. This is with two notable exceptions: the union of the unikonts and, much more interestingly, the strongly supported (BP = 100%; BiPP = 1.0) assemblage of stramenopiles, Rhizaria, and alveolates (clade SAR), with these last two groups being robustly clustered together (BP = 88%; BiPP = 1.0) (clade SR). Comparisons of substitution rates between the different lineages were highly non significant at 1.25%, indicating that all species evolve at very similar rates, thus rendering unlikely a possible artefact caused by long branches (data not shown).

To further test this unexpected nested position of Rhizaria between alveolates and stramenopiles, we compared different topologies by performing the approximately unbiased (AU) test, which is considered as the least-biased and most rigorous test available to date [30]. More precisely we evaluated two questions: 1) Are Rhizaria indeed monophyletic with stramenopiles and alveolates; 2) Are Rhizaria specifically related to stramenopiles, with the exclusion of alveolates? Our analyses show that an alternative topology, which corresponded to the best topology with Rhizaria forced not to share a common ancestor with the assemblage composed of stramenopiles and alveolates (Figure S3; Table 1B), had a likelihood significantly lower than the best ML tree obtained without constraint (Figure 1; Table 1A) at the significance level of 0.05 (*P* = 4e-008). On the other hand, the two other possible positions for Rhizaria within the SAR grouping (Table 1D, E) could not be significantly rejected (*P* = 0.112; *P* = 0.079, respectively), thus preventing the exclusion of a specific relationship between Rhizaria and alveolates or an early divergence of Rhizaria. In addition, we also tested the relationship between Rhizaria and excavates by evaluating all possible trees in which these two groups are monophyletic. None of these trees could be retained in the pool of plausible candidates (data not shown).

**Table 1:** Likelihood AU Tests of Alternative Tree Topologies.

Tree topology	A	B	C	D	E
	Fig. 1	Fig. S3	A(RS)	R(SA)	S(RA)
Au^a^	1.0	4e-008	0.895	0.112	0.079
Δ ln L^b^	−369.2	369.2	−27.4	69.4	77.5

A, B) Comparison between topology A (best tree, corresponding to the Figure 1) and the alternative topology B (corresponding to the best tree when Rhizaria are forced not to be monophyletic with S and A, Figure S3).

C, D, E) Comparisons between topology C (best tree) and the alternative topologies D and E.

Abbreviations are as follows: A = alveolates; S = stramenopiles; R = Rhizaria

Underlined number corresponds to the significant *P* value of the rejected topology.

a Approximate Unbiased Test.

b Log likelihood difference.

## Discussion

We present in this study the largest dataset currently available for eukaryote phylogeny combining both an extensive taxa sampling and a large amount of amino acid positions. Our analyses of this unique dataset bring a strong evidence for the assemblage of Rhizaria, stramenopiles and alveolates. Therefore we propose to label this monophyletic clade ’SAR’. Although weakly suggested in our previous multigene analysis [19], we show here using a much larger dataset that this specific grouping is in fact very robust. We confirm the existence of consistent affinities between assemblages that were thought to belong to different supergroups of eukaryotes, thus not sharing a close evolutionary history. The addition of about 20 relevant taxa of unicellular eukaryotes as well as more than 30 genes (to a total of 123 genes) seems to have stabilized the topology to consistently display a monophyly of SAR. Within this newly emerged assemblage, Rhizaria appear to be more closely related to stramenopiles than to alveolates, but topology comparisons failed to discard alternative possibilities (i.e. R(SA) or S(RA)). In addition, we clearly reject the putative relationship between Rhizaria and excavates [16], [19], which has been already convincingly tested in [31].

Interestingly, an association between Rhizaria and stramenopiles could already be observed in 18S rRNA trees representing a very large diversity of eukaryotes (see for example [32]–[34]). More recently, the analysis of 16 protein sequences from 46 taxa also showed a robust clade consisting of Rhizaria, alveolates, and stramenopiles [29]. However, this work significantly differs from ours by rejecting the association of Rhizaria as sister to stramenopiles or as sister to all chromalveolates. Beside our much larger dataset, it is unclear why our data display more flexibility with respect to the position of Rhizaria within the SAR monophyletic clade. More comprehensive taxa sampling for both Rhizaria and stramenopiles, particularly for early diverging species (e.g. radiolarians), is likely to shed light on the internal order of divergence within SAR.

These new relationships suggest that the supergroup ‘Chromalveolata’, as originally defined [16], does not correctly explain the evolutionary history of organisms bearing plastids derived from a red algae. In fact, our results confirm the lack of support chromalveolates as a whole (i.e. including haptophytes and cryptophytes) received in several studies [3]. The phylogenetic position within the eukaryotic tree of the monophyletic group haptophytes+cryptophytes is uncertain [13]. Globally, chromalveolates have been strongly supported by phylogenies of plastid genes and unique gene replacements in these taxa [35]–[37], but the monophyly of all its members has never been robustly recovered with nuclear loci, even using more than 18000 amino acids (Patron et al. 2007). Overall, the unresolved nodes between the chromalveolates lineages have prevented clear conclusions relative to this model of evolution [3], [15].

The emergence of SAR may potentially complicate the situation of secondary endosymbioses and questions the most parsimonious explanation of the evolution of chlorophyll-c containing plastids (see also [19], [29], [38], [39]). At this stage at least two scenarios are conceivable, but none of them can be presently favoured by concurrent topologies due to the uncertain position of the haptophytes and cryptophytes clade. First, a single engulfment of red algae might have occurred in a very early stage of chromalveolates evolution and the resulting plastid was secondarily lost in certain lineages, such as ciliates and Rhizaria. Second, it is possible that stramenopiles (or alveolates, or even haptophytes+cryptophytes, depending on their real position within the tree) have acquired their secondary plastid in an independent endosymbiosis event from a red algal organism. If this latter scenario is correct, minimizing the number of endosymbiosis events as proposed by the chromalveolates hypothesis might actually not correspond to the true symbiogenesis history. So far, as many as 11 primary, secondary, and tertiary symbiotic events have been identified (see [12]). Notably, two independent secondary endosymbiosis events involving green algae have been recognized in members of excavates and Rhizaria: Euglenozoa and chlorarachniophytes [31], respectively. Hence, multiplying the number of secondary endosymbiosis might better explain the phylogenetic relationships within eukaryotes than the chromalveolate hypothesis.

The new SAR supergroup implies that the major part of protists diversity shares a common ancestor. Indeed, the chromalveolates members alone already accounted for about half of the recognized species of protists and algae [40]. With the addition of rhizarians, a huge variety of organisms with very different ecology and morphology are now united within a single monophyletic clade. Finding a synapomorphy that would endorse the unification of these groups will be the next most challenging step in the establishment of eukaryote phylogeny.

## Materials and Methods

### Sampling, culture and construction of cDNA libraries

The miliolids of genus *Quinqueloculina* were collected in the locality called Le Boucanet, near La Grande Motte (Camargue, France). They were sorted, picked, and cleaned by hand under the dissecting microscope. The culture of *G. cometa* was taken from the culture collection of IBIW RAS (Russia) and maintained as described in [20]. Cells were collected by low-speed centrifugation, resuspended into five volumes of TriReagent (Invitrogen, Carlsbad, Calif.), and broken using manual pestles and adapted microtubes. Total RNA and cDNA were prepared as in [21]. EST sequencing of the *Quinqueloculina* sp library was performed with the ABI-PRISM Big Dye Terminator Cycle Sequencing Kit and analysed with an ABI-3100 DNA Sequencer (Perkin-Elmer Inc., Wellesley, Mass.), all according to the manufacturer's instructions. The *G. cometa* library was sequenced by Agencourt Bioscience Corporation (Beverly, Mass.).

### Construction of the alignments

We performed TblastN searches against GenBank using as queries a rhizarian dataset made of all translated sequences (translations done with transeq, available at the University of Oslo Bioportal; http://www.bioportal.uio.no) for *R. filosa*, *Quinqueloculina* sp., *G. cometa*, and *B. natans*. We retrieved and translated all sequences with an *e*-value cutoff at 10^−40^, accounting for 46 new genes out of a total of 126. The rest of the genes (i.e. 80 genes) corresponded to rhizarian proteins putatively homologous to sequences previously used to infer large-scale phylogenies [41] and available at http://megasun.bch.umontreal.ca/Software/scafos/scafos_download.html. In order to roughly check for orthology, we also added to these alignments the human sequence with the lowest *e*-value in our TblastN output to make sure that no closer homologs were known. These 126 genes were used to build a very well-sampled dataset by adding all available relevant species. For this purpose, we considered all species in TBestDB as well as all other bikont taxa for which sufficient sequence data were available and made a local database against which we ran TblastN searches with our rhizarian dataset (*e*-value threshold 10^−40^).

To decide on the final set of genes used in this study, we carefully tested the orthology for each of the 126 selected genes by carrying out Maximum likelihood (ML) analyses including bootstrap supports with the program TREEFINDER (JTT, 4 gamma categories and 100 bootstrap replications) [42]. For three genes, the overall orthology could not be assessed with enough confidence and thus were removed. More generally, taxa displaying suspicious phylogenetic position were removed from the single-gene dataset. Once this pre-screen was complete, our final taxon sampling comprises 49 species and 123 genes (Table S1). We concatenated all single gene alignments into a supermatrix alignment using Scafos [43]. Because of the limited data for certain groups and to maximize the number of genes by taxonomic assemblage, some lineages were represented by different closely related species always belonging to the same genus (for details see Tables S2 and S3).

### Phylogenomic analyses

The concatenated alignment was first analyzed using the maximum likelihood (ML) framework encoded in TREEFINDER, with the global tree searching procedure (10 starting trees) [42]. In order to double-check our topologies, we also ran RAxML (RAxML-VI-HPC-2.2.3) [44], using randomized maximum parsimony (MP) starting trees in multiple inferences and the rapid hill-climbing algorithm. Following the Akaike Information Criterion (AIC) [45] computed with ProtTest 1.3 [46], the RtREV+G+F model allowing between-site rate variation was chosen (calculations were done with 6 gamma categories). The WAG model was also tested and gave the same topologies. To estimate the robustness of the phylogenetic inference, we used the bootstrap method [47] with 100 pseudoreplicates in all analyses.

Bayesian analysis using the WAG+G+F model (4 gamma categories) was preformed with the parallel version of MrBayes 3.1.2 [48]. The inference, starting from a random tree and using four Metropolis-coupled Markov Chain Monte Carlo (MCMCMC), consisted of 1,000,000 generations with sampling every 100 generations. The average standard deviation of split frequencies was used to assess the convergence of the two runs. Bayesian posterior probabilities were calculated from the majority rule consensus of the tree sampled after the initial burnin period as determined by checking the convergence of likelihood values across MCMCMC generations (corresponding to 50,000 generations, depending on the analysis).

The evolutionary rates of the selected species were calculated with the relative-rate test as implemented in RRTree [49], by doing pairwise comparisons of two ingroups belonging to either SAR, hatptophytes+cryptophytes, excavates or plants relatively to the unikonts taken as outgroup.

### Tree topology tests

To better assess the phylogenetic position of Rhizaria, we conducted topology comparisons using the approximately unbiased (AU) test [30]. For each tested tree, site likelihoods were calculated using CODEML and the AU test was performed using CONSEL [50] with default scaling and replicate values. To test the monophyly of the new assemblage SAR, we first compared our tree (Figure 1) to the best possible tree in which Rhizaria were forced to be outside SAR, given topological constraints corresponding to a trichotomy of unikonts, stramenopiles+alveolates, and the rest of the groups represented as a multifurcation (Figure S3). Secondly, we evaluated the placement of Rhizaria within the SAR clade by testing the three possible branching patterns between Rhizaria, stramenopiles, and alveolates.

## Supporting Information

Figure S1 Best RAxML tree of eukaryotes.Numbers at nodes represent the result of the bootstrap analysis; black dots mean values of 100% (hundred bootstrap replicates were done). Nodes with support under 65% were collapsed.
(3.29 MB TIF)

Figure S2 MrBayes tree. Numbers at nodes represent the bayesian posterior probabilities.
(3.37 MB TIF)

Figure S3 Best TREEFINDER tree in which Rhizaria were forced not to belong to SAR.
(3.34 MB TIF)

Table S1 Abbreviated and complete protein names.
(0.05 MB XLS)

Table S2 OTU (Operational Taxonomic Unit) names, number of characters, and percentage of characters included in the final alignment
(0.02 MB XLS)

Table S3 Percentage of missing data per species and per genes
(0.06 MB XLS)
